# Haplogrep 3 - an interactive haplogroup classification and analysis platform

**DOI:** 10.1093/nar/gkad284

**Published:** 2023-04-18

**Authors:** Sebastian Schönherr, Hansi Weissensteiner, Florian Kronenberg, Lukas Forer

**Affiliations:** Institute of Genetic Epidemiology, Medical University of Innsbruck, Innsbruck 6020, Austria; Institute of Genetic Epidemiology, Medical University of Innsbruck, Innsbruck 6020, Austria; Institute of Genetic Epidemiology, Medical University of Innsbruck, Innsbruck 6020, Austria; Institute of Genetic Epidemiology, Medical University of Innsbruck, Innsbruck 6020, Austria

## Abstract

Over the last decade, Haplogrep has become a standard tool for haplogroup classification in the field of human mitochondrial DNA and is widely used by medical, forensic, and evolutionary researchers. Haplogrep scales well for thousands of samples, supports many file formats and provides an intuitive graphical web interface. Nevertheless, the currently available version has limitations when applying it to large biobank-scale data. In this paper, we present a major upgrade to the software by adding (a) haplogroup summary statistics and variant annotations from various publicly available genome databases, (b) an interface to connect new phylogenetic trees, (c) a new state-of-the-art web framework managing large scale data, (d) algorithmic adaptions to improve FASTA classification using BWA-specific alignment rules and (e) a pre-classification quality control step for VCF samples. These improvements will give researchers the opportunity to classify thousands of samples as usual but providing additional ways to investigate the dataset directly in the browser. The web service and its documentation can be accessed freely without any registration at https://haplogrep.i-med.ac.at.

## Graphical Abstract

**Graphical Abstract:**
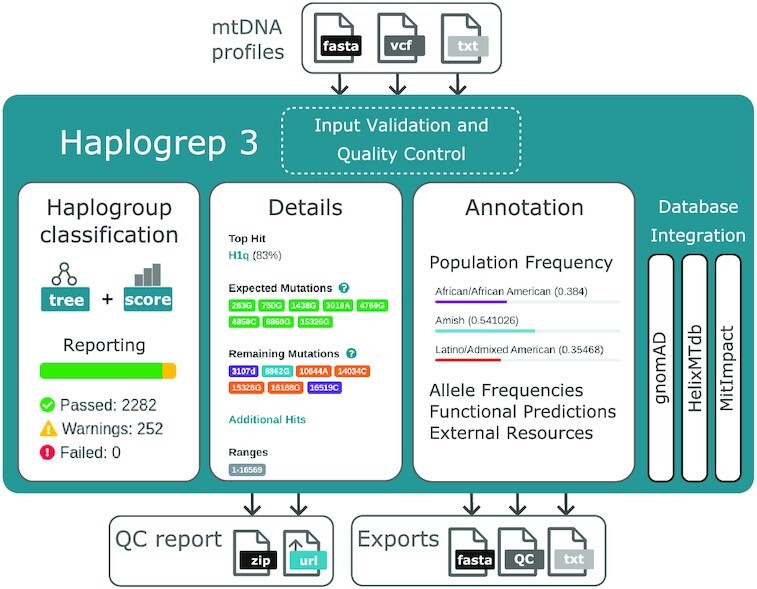
Haplogrep 3 allows the upload of mitochondrial profiles in different formats for haplogroup classification and also includes steps for input validation or quality control. The user interface reports summary statistics and provides annotations for each sample.

## INTRODUCTION

Available phylogenetic presentations of sequenced samples (so called phylogenetic trees) allow to classify uniparentally transmitted haplotypes into haplogroups (1). Each phylogenetic haplogroup (or branch) within the tree is identified by a unique set of variants differing from a reference genome. The currently most updated phylogenetic tree for human mitochondrial genomes includes 6401 unique haplogroups (2), which is based on the well-known phylogenetic tree Phylotree (3). Previously, we developed Haplogrep to assign mitochondrial input profiles the best matching haplogroup in an automated way. Haplogrep works by traversing a phylogenetic tree, calculating the distance to each haplogroup using the Kulczynski metric and returning a sorted list of haplogroup hits for each sample (4). In 2016, we improved the software by integrating a rule-based system assigning each sample a quality status, supporting new file formats and providing a command-line version, which has also been integrated in several workflow pipelines and online services (5). The set of included features, frequent updates of supported phylogenetic trees and our community support made Haplogrep to one of the most accurate and widest used tools for haplogroup classification in mtDNA studies (6).

Nevertheless, over the last years we also identified shortcomings with the currently available version: first, genomic databases like gnomAD (7) provide a rich set of annotations such as variant or population frequencies for evaluating sites of interest. Many of these necessary downstream-analysis steps are currently not directly supported within the application, making it unnecessarily complicated for researchers to analyse samples initially classified with Haplogrep. Second, modern technologies allow sequencing thousands of samples with decreasing costs, therefore making biobank-scale datasets available to researchers. While Haplogrep's classification algorithm works well for many samples, the web application was not developed for screening thousands of samples directly in the browser and does not provide any summary statistics on the uploaded dataset. Third, while Haplogrep already supports numerous phylogenetic trees, the integration of new trees is closely bundled to the software itself, making it complex to add new trees for external users. Forth, input data is nowadays coming from heterogeneous resources (e.g. genotyping arrays (8), whole-genome sequenced samples (9), Sanger-sequenced data (10), long-read data (11)), which requires a systematic quality control before haplogroup classification to avoid unexpected pitfalls.

To address these shortcomings, we developed Haplogrep 3. It includes all key features from previous versions, eliminates the mentioned shortcomings and is available as a hosted web service but can also be run graphically or on the command-line in a private environment. This version will greatly improve how researchers analyse datasets, allowing them identifying input errors or spurious results as early as possible.

## MATERIALS AND METHODS

### Webserver

Haplogrep 3 is a web application developed in Java using the Javalin web framework in combination with a template engine for Java. For visualization, we use the D3.js and Chart.js JavaScript libraries. The main algorithm of Haplogrep has been capsulated into a library (haplogrep-core), integrated into the application as a dependency and has already been described in detail elsewhere (4). Haplogrep 3 provides several sub-commands (server, classify, distance, trees, install-trees) to end users, which allows to run it locally or integrate it in automated pipelines. The *server* command starts a new web-server instance, *classify* allows to run haplogroup classification on the command line, *distance* calculates the distance between two input haplogroups, *trees* returns the currently installed trees and *install-trees* allows to install new phylogenetic trees. Unlike to previous versions, the same code base is now used for the web application and the command-line tool. Haplogrep 3 loads all required information from a configuration file in YAML syntax. This file includes basic features like upload limit, port, provided test datasets but also includes the currently installed phylogenetic trees and the location of the phylogenetic trees' repository.

### Integration of phylogenetic trees

Starting with Haplogrep 3, phylogenetic trees are now hosted in a separate repository (https://genepi.github.io/haplogrep-trees). Each tree includes a set of required files for the integration into Haplogrep. It consists of (a) the tree in XML syntax, (b) variants weights for haplogroup classification, (c) hotspot locations not considered for phylogenetic interpretation, (d) annotation files from external databases, (e) reference sequence (e.g. rCRS (12) or RSRS (13)) and required BWA files and (f) a set of FASTA alignment rules. This architectural change allowed us to decouple Haplogrep from a specific phylogenetic tree and will simplify the integration of new trees in future releases. All installed trees are loaded at startup and user can select one of the trees before classification starts.

### FASTA improvement and alignment rules

To support FASTA as an input format, we integrated the BWA alignment software (14) as a JNI library. Unlike to sequence alignment with BWA where insertions and deletions (indels) are left aligned, the currently available mitochondrial phylogenetic trees expect right aligned indels. Therefore, indels are not correctly placed by BWA and result in a lower overall haplogroup quality or even misclassification. To adjust for that, we integrated a set of currently 123 nomenclature rules that are applied by default prior to haplogroup classification. The rules have been generated by comparing expected and remaining variants from haplogroup-defining samples from the updated Phylotree (2) in FASTA format. By comparing these variants for each input sample, we were able to create a list of rules which fix issues especially for indel alignments. The list of rules is included as a file in each phylogenetic tree, making it adaptable in future releases. Besides that, Haplogrep 3 has been improved to work with partial FASTA sequences (e.g. control region), a previous shortcoming of the software.

### Haplogroup clustering and search

Haplogrep 3 introduces a new clustering of haplogroups using the top-level haplogroups (or clusters) as defined by Phylotree and gnomAD. For each input sample or defining haplogroup of an available tree, we calculate the distance to each of the 33 top-level haplogroups and use the cluster with the minimal distance as a result. Using this new tree structure, users can search for haplogroups and variants directly within the application.

### Variant annotation

For variant annotation, we used external data from gnomAD (7), MitImpact (15) and the Helix Mitochondrial database (16), which have been integrated in the application. The files have been downloaded and indexed for accessing variant details in real-time. Each provided phylogenetic tree package also consists of a version number, which allows us to integrate possible future database updates in a reproducible way.

## RESULTS

### Analysis workflow

**Figure 1.**
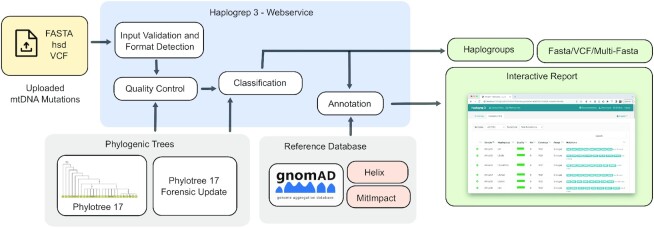
Analysis workflow of Haplogrep 3. Haplogrep allows the upload of hsd, FASTA or VCF files. Initially, the samples are checked and validated. Before haplogroup classification, a quality control step is executed to provide summary statistics on the uploaded file. Haplogroups are then calculated and enriched with numerous annotations on a haplogroup and variant level. All results are displayed in the web-browser for further investigation.

Like previous versions, users can upload their sample data in a text format (hsd), as FASTA or in the variant calling format (VCF). The server assigns each sample the top haplogroup hits (currently 20) by traversing through the selected phylogenetic tree. The input format of the samples is autodetected by the software but can also be set manually. This is especially useful for VCF files, where users can specify if input files originate from genotyping arrays or how heteroplasmic positions from next-generation sequencing data should be handled by the system. If the samples come from genotyping arrays, Haplogrep 3 automatically adapts the search range to the available positions included in the VCF file. Before classification, users can select one of the available Haplogrep metrics on how distances to haplogroups are calculated, the phylogenetic tree and if additional output formats should be generated. Haplogrep then executes a quality control (QC) step and calculates summary statistics for all samples. After the actual haplogroup classification, an annotation step is executed and users are forwarded to the results including a summary dashboard for visual analytics (e.g. data grouped by top-level haplogroups including population information, QC statistics) and the sample details (see Figure 1).

### Summary dashboard

Haplogrep 3 includes a graphical summary dashboard including statistics regarding the sample classification. It consists of a graphical overview of all individual haplogroups and their distribution into the 33 top-level haplogroups (see Materials and Methods). The dashboard also consists of a table of all uploaded samples showing the population composition for each top-level haplogroup with ancestry information provided by gnomAD. Besides that, it also includes all available export formats including the possibilities to download the sample QC report as a tab-delimited file. Unlike to previous versions, export formats are generated on-the-fly during classification and can be downloaded without any delay.

### Sample overview

Besides the summary dashboard, the individual samples page includes details for each sample (e.g. number of expected variants, found variants, additional variants), the top 20 hits for each input sample and the analysed range. To investigate samples, Haplogrep 3 allows to analyse each variant associated with a haplogroup by clicking on it. This will allow users to access variant or population frequencies and evaluate variants in more detail (see ‘Interactive Variant Discoveries’).

### Phylogenetic trees

Haplogrep 3 supports the integration of external phylogenetic trees. We currently provide a set of five mtDNA trees within Haplogrep 3, all managed in separate Git repositories for reproducibility (https://genepi.github.io/haplogrep-trees). Each tree consists of a configuration file and a set of annotation files (see Material and Methods). This architectural change allowed us to decouple Haplogrep from the phylogenetic trees and simplifies the integration of new trees for external users. While all trees are currently mtDNA specific, Haplogrep 3 is not limited to mtDNA allowing other phylogenetic representations to benefit from its features. Beginning with this version, we also provide a new feature to make phylogenies searchable for end users. Users can use Haplogrep 3 to navigate through all top-level haplogroups and access textual and graphical representations for each haplogroup including expected variants.

### VCF pre-classification quality control

VCF files can originate from different projects (e.g. microarray projects, whole-exome/whole-genome or long-read sequencing projects) and are often missing an external quality-control step before classification. This can result in a lower haplogroup quality or even in a misclassification. The latest version of Haplogrep now integrates an initial validation step for VCF samples and includes (a) an analysis of the uploaded sample file (e.g. number of samples, number of variants, overlap with the phylogenetic tree, monomorphic variants), (b) a check on possible strand-flips and (c) statistics on the variant or sample call rate.

### Interactive variant discoveries

Each phylogenetic tree includes a set of annotation files. Haplogrep 3 displays all available information directly in the web-application, when a specific variant is selected (see Material and Methods). Access to variant frequencies allow users to evaluate each variant in detail and assess its clinical relevance using functional predictors from MitImpact (15). Haplogrep 3 also provides direct links to external resources for further investigation and allows to export a set of predefined annotations.

### Analysing 1000 genomes data with Haplogrep 3

**Figure 2.**
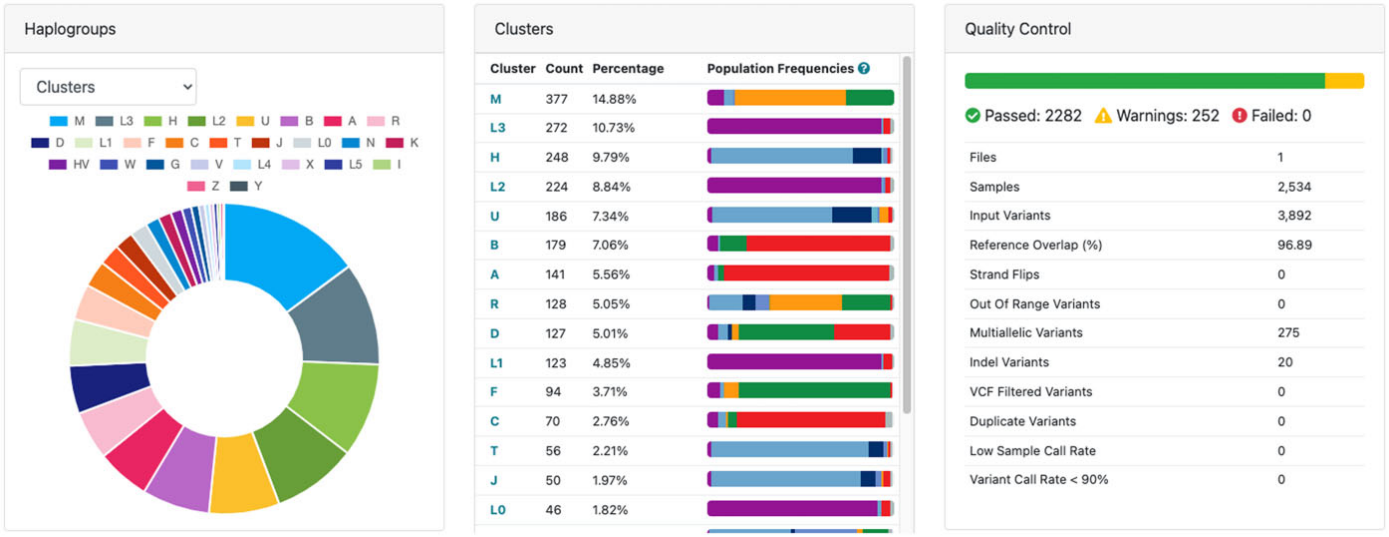
Summary dashboard of haplogrep 3. The dashboard shows the overview of 2534 samples from the 1000 Genomes Phase 3 project. 89% of the samples (2282) include no warnings, 11% show a warning which needs further investigation in the Sample tab. The clusters table includes all found haplogroups summarized by top-level haplogroup and annotated with the population frequencies from gnomAD. The report can be downloaded or shared via a link.

The new web application allows to investigate samples in more depth directly in the browser. Here, we are analysing the 1000 Genomes data, which have been made available by the 1000 Genomes Project Phase 3 (17) and includes a set of 2534 samples. All samples have been classified in 88 seconds on the publicly available web service using the default parameters (see Supplementary Table S1). The newly created dashboard shows an overview of all samples and indicated that all samples are passing QC. Samples are clustered by top-level haplogroup or by individual haplogroup. All 33 clusters as defined by Phylotree are represented. 11% of the uploaded samples include a warning, mainly because of a slightly decreased haplogroup quality (see Figure 2).

**Figure 3.**
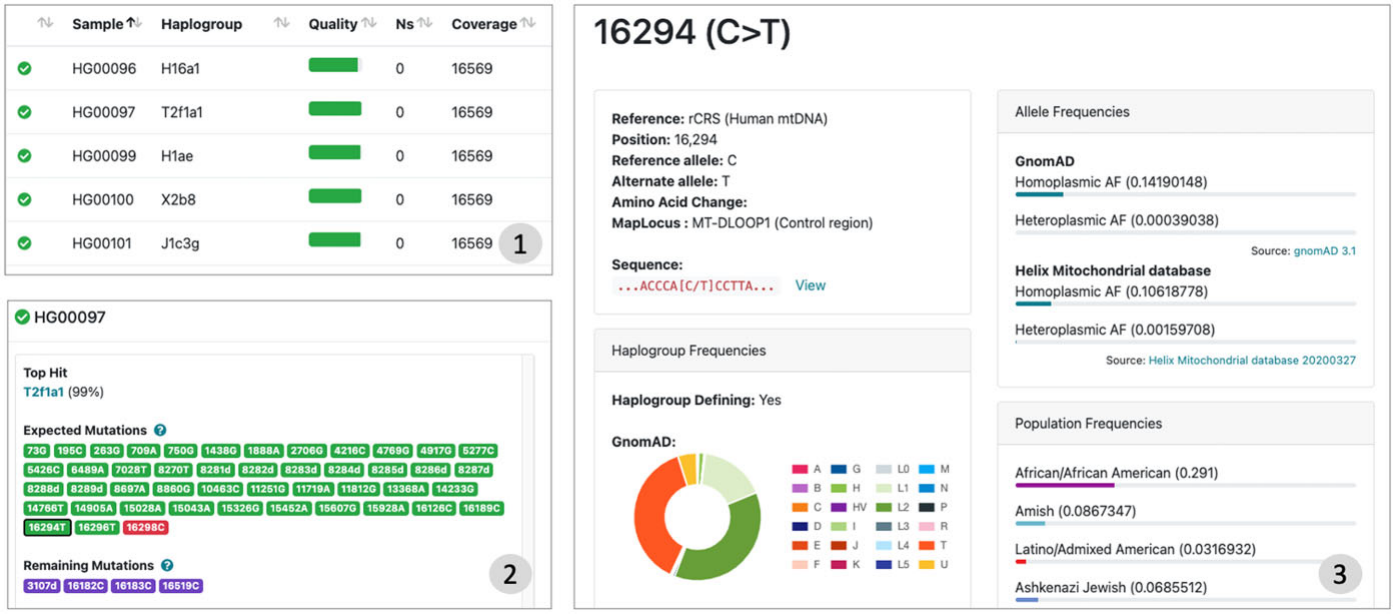
(1) All samples are provided as a table including the most important information like detected haplogroup, quality score or position coverage. (2) By selecting a sample, additional information is displayed showing all excepted and remaining variants for the top hit haplogroup. (3) Each variant can be selected to display numerous frequencies and predictions.

The details tab of Haplogrep 3 shows how a sample report is structured. It includes the haplogroup top hit, expected mutations of the haplogroup and remaining mutations of each sample, the top 20 additional haplogroup hits and the analysed sample range. It also includes frequencies for each variant (provided by publicly available databases) for further investigation (see Figure 3).

## DISCUSSION

Published in 2010 and 2016, Haplogrep was the first fully automated haplogroup classification tool for mtDNA. The number of citations and downloads shows that Haplogrep is a critical tool for numerous research areas and supports studies at any size. With the upgrade presented in this paper, we go a step further and provide users new tools for evaluating variants and new possibilities to look at their data. We also improved the FASTA alignment for partial samples, added a new QC step for VCF samples and decoupled the phylogenetic trees from the application itself. Since our original publication, many tools have been published showing a similar set of features or provided functionally which were initially not available in Haplogrep. Nevertheless, none of the currently available tools include the same set of features compared to Haplogrep. HaploGrouper (18) works for any kind of phylogenetic trees but supports only VCF and is not available as a graphical web service. MitoSuite (19) requires a local installation and works only for next-generation sequencing data in BAM format. HaploTracker (20) works well for partial FASTA sequences, a feature which has also been integrated in Haplogrep 3. HaploCart1.0 (21) uses a novel and promising pangenome reference structure eliminating problems when the sample is identical to the rCRS but is currently too computational expensive to run on a large set of samples. MitoSuite (22) uses Haplogrep for mtDNA classification but also includes several tools to further investigate variants. Many of these annotations are now also available within Haplogrep 3.

While the integration of new trees is now simplified, the creation of phylogenetic trees can pose numerous challenges to end users. Since Haplogrep is a constantly developing service since >10 years, we are planning to provide automated ways to create the required files, which can then be connected to Haplogrep. Overall, we think that the new set of features will greatly improve and simplify the work with Haplogrep and provide users for the first-time functionality for downstream analysis directly within the application.

## DATA AVAILABILITY

The Haplogrep 3 webserver is available at: https://haplogrep.i-med.ac.at. The Haplogrep 3 source code is available at: https://github.com/genepi/haplogrep3 and https://doi.org/10.5281/zenodo.7801633. The Haplogrep 3 documentation including examples is available at: https://haplogrep.readthedocs.io.

## Supplementary Material

gkad284_Supplemental_File
